# Identification of the Eph receptor pathway as a novel target for eicosapentaenoic acid (EPA) modification of gene expression in human colon adenocarcinoma cells (HT-29)

**DOI:** 10.1186/1743-7075-7-56

**Published:** 2010-07-12

**Authors:** Joanne F Doleman, John J Eady, Ruan M Elliott, Rob J Foxall, John Seers, Ian T Johnson, Elizabeth K Lund

**Affiliations:** 1 Institute of Food Research, Norwich Research Park, Colney, Norwich, NR4 7UA, UK

## Abstract

**Background:**

The health benefits of polyunsaturated fatty acids (PUFAs), particularly those of the n-3 series are well documented. The mechanisms by which these effects are mediated are not fully clarified.

**Methods:**

We used microarrays to assess the effects on gene expression in HT29 colon adenocarcinoma cells of exposure to the n-3 fatty acid eicosapentaenoic acid (EPA). HT29 cells were cultured with EPA (150 μM) for up to 24 hr prior to harvesting and isolation of RNA. Microarray results were analyzed within the statistical package 'R', and GeneGo MetaCore was used to identify key pathways of altered gene expression.

**Results:**

EphB4, Vav2 and EphA1 gene expression were identified as significantly altered by EPA treatment. Statistically significant changes in gene expression after HT29 exposure to EPA were confirmed in a second experiment by real-time RT-PCR (TaqMan), This experiment also compared the effects of exposure to EPA to arachadonic acid (AA, n-6). Corresponding changes in protein expression were also assessed by Western blotting.

**Conclusions:**

Eph receptor mediated signaling is an entirely novel signaling pathway through which EPA may promote a wide range of health benefits, in particular in relation to reduction of colorectal cancer progression.

## Background

Polyunsaturated fatty acids (PUFAs), and in particular those of the n-3 series found in fish oil, are well recognized to have a wide range of health benefits [1]. However the mechanisms by which they mediate their beneficial physiological effects at the cell signaling level are still poorly understood. The importance of PUFAs in modulating cell function via control of gene transcription has been recognized for over a decade [2]. The potential complexity of the various signaling mechanisms involved when cells are exposed to PUFAs is discussed in a review by Tang *et al*. [3]. For example they, and the wide range of signaling molecules derived from them, act as ligands to PPARs. They may also change the fluidity of cell membranes and thus influence receptor activity, and they can also modify the redox state, which in turn will influence a wide range of signaling pathways. It is entirely feasible to raise plasma and tissue fatty acids to levels which have been shown to be biologically active *in vitro *by taking fish oil capsules [4], and probably by consuming oil-rich fish [5,6]. For example, in volunteers consuming 3 g fish oil per day for 18 weeks, plasma concentrations of the two long chain n-3 fatty acids eicosapentaenoic acid (EPA; C20:5) and docosahexaenoic acid (DHA; C22:6) reached concentrations in excess of 300 μM [4]. Similarly Gee *et al*. have shown that consuming fish oil capsules leads to a significant increase in EPA concentration in colon biopsy tissue [7].

The potential importance of dietary n-3 fatty acids in relation to colorectal cancer prevention has become increasingly well recognized over the last decade. Epidemiological studies have shown that high fish consumption is associated with a reduced incidence of colorectal cancer [8] and *in vitro *studies have provided evidence of potential mechanisms. Animal studies, using a chemical model of colorectal cancer, have shown that dietary fish oil both reduces cell proliferation and induces apoptosis [9,10]; changes which are associated with a reduction in the formation of aberrant crypt foci. In humans, consumption of n-3 fatty acids has been shown to reduce crypt cell proliferation rate in the colon and rectum of patients at risk of developing colorectal cancer [11] and this finding is associated with a reduction in the number of polyps [12]. Additionally these highly unsaturated fatty acids have been shown to induce apoptosis in cultured colorectal carcinoma cells at concentrations within the range found in plasma. While both EPA and DHA have been shown to have anticarcinogenic effects, previous studies in our group have specifically shown effects of EPA on tumor suppression [13]. Although a number of potential mechanisms by which fish oil derived fatty acids may protect against colorectal cancer have been suggested including modifying gene expression either through response to oxidative stress or via PPAR signaling [14-17], the molecular mechanism by which a high-fish diet, mediates these effects needs clarification. The aim of this study was to identify novel pathways by which the n-3 PUFA, EPA, may modify tumor progression.

We have used microarray analysis to make an initial assessment of changes in expression of genes in HT29 colon adenocarcinoma cells over a 24 hr period following exposure to EPA. This timescale was chosen as a result of an initial screening study which showed that EPA treatment was linked to modified redox potential within the cell, as measured by the ratio of oxidized to reduced glutathione, such that this ratio was doubled from 2-4 hr post exposure, before returning to baseline. Meanwhile total glutathione levels showed a steady decline to about 50% of the initial value over 24 hr (unpublished data). In the present study, pathway analysis was carried out to assess which pathways were modulated by EPA treatment. This identified genes involved in ephrin (Eph) receptor signaling as of key importance.

In order to assess the specificity of the effects of different PUFA classes on this signaling pathway we conducted a second experiment, using real-time RT-PCR to investigate changes over time, in those genes identified by microarray. In this experiment HT29 cells were exposed to either EPA or arachadonic acid (AA), relative to changes in control cells not exposed to PUFA. Exposure to AA was used to compare the effects of a highly unsaturated n-3 PUFA (EPA, C20: 5) with a similarly unsaturated n-6 PUFA (AA, C20:4). If the effects of PUFA were associated purely with changes in redox potential it might be expected that both fatty acids would show similar effects, whereas if they act as ligands for nuclear receptors they may be expected to cause different effects [18]. Finally the changes identified at the gene level have then been assessed at the protein level by western blotting.

## Methods

### Cell culture

The human colonic epithelial cells were obtained from European collection of cell cultures (ECACC). The cells were cultured under standard culture condition in Dulbecco's modified Eagle's medium (DMEM) (Sigma) supplemented with 10% fetal bovine serum, 2 mM L-glutamine and 1% penicillin-streptomycin. The cells were maintained at 37°C and 5% CO_2 _in a humid environment.

### Treatment of cells

Two separate experiments were carried out. The first experiment focused on identifying novel signaling pathways associated with exposure to EPA using microarray analysis. The second was designed to confirm results found by array analysis and to compare with the effects of an n-6 polyunsaturated fatty acid AA. EPA (E-2011) and AA (A-9673) were purchased from Sigma and diluted in ethanol to 25 mg/ml. These stocks were stored at -20°C under nitrogen to prevent oxidation. 75 cm^2 ^flasks were seeded with 6 × 10^6 ^cells in 25 ml medium and left to adhere for 24 hr. After this time the medium was removed and replaced with either 25 ml fresh medium (control) or 25 ml medium containing EPA (EPA treated) or 25 ml medium containing AA (AA treated) at a final concentration of 150 μM. The medium contained 0.2% ethanol which is sufficiently low to disregard solvent effects. This concentration was chosen as a result of preliminary experiments showing this was the maximum that did not lead to reduction in cell number, over 24 hr incubation as shown by neutral red cell viability assay [19]. Four flasks of control cells and four flasks of EPA treated cells were harvested with trypsin within 20 min of the media change (t = 0 hr) and then at 1, 2, 4, 6, 8, and 24 hr for microarray analysis. For real-time RT-PCR analysis, 3 flasks of EPA, AA and untreated cells were harvested at all the time points. For protein analysis, 3 flasks of each treatment (EPA, AA and untreated) were harvested immediately and at 2, 6, 8 and 24 hr. Cells were stored as a dry pellet at -80°C prior to extraction of RNA or protein. The cells for microarray analysis were grown and treated on different dates to those grown and treated for real-time RT-PCR and protein analysis.

### RNA extraction

Total RNA was extracted using Qiagen RNeasy mini and midi kits as appropriate. The eluted total RNA was stored at -80°C. The concentration and quality of the RNA was determined by Agilent lab-on-a-chip RNA 6000 nano chips. Accuracy of concentration was checked with a subset of samples on a spectrophotometer. A large pool of HT29 RNA was also extracted for use as a reference RNA sample for the microarray analysis. For real-time RT-PCR analysis, the integrity of all RNA samples was confirmed using the Agilent lab-on-a-chip bioanalyser and the RNA concentrations were determined using the nano drop ND-1000 spectrophotometer.

### Experiment 1: Microarray analysis

Cy3 (test sample) and Cy5 (reference sample) labeled cDNA extracts were prepared by reverse transcription of the RNA samples obtained from the HT29 cells in the presence of amino-allyl dUTP to enable subsequent coupling with the Cy dyes (GE Healthcare Life Sciences) using a well established protocol [20]. The labeled cDNA samples were hybridized to in-house printed oligonucleotide arrays as described previously [21]. The array consists of 13,971 gene-specific oligonucleotides, 29 negative controls and 1 positive control (an equimolar mixture of the 13,971 gene-specific oligonucleotides). The arrays were scanned using an Agilent G2565BA microarray scanner system (Agilent Technologies). The raw signal intensities for the features on the microarrays were extracted using Axon GenePix 4.0 software (Axon Instruments). Bad features, identified by visual inspection, were flagged as such manually. All microarray data has been deposited into ArrayExpress public repository. Accession code: E-MEXP-2673.

Subsequent data analyses were restricted to features flagged automatically by the software as present. Array data analysis was performed using R http://www.R-project.org and GeneGo MetaCore (GeneGo bioinformatics software, Inc.).

Raw un-normalized array data were uploaded into the statistical package 'R', using the Limma package, and bad flagged spots were omitted. Data were normalized with Lowess print tip normalization and, as a standard reference sample was used in the Cy5 channel, normalization between arrays was performed using the GQuantile approach. Differences between test and control at specific time points were determined, taking into account the differences at baseline T0. Fold change data was determined for the data with an adjusted p value of < 0.05 with a Benjamini Hochberg multiple test correction factor.

The ID, unigene number, adjusted p value and fold change data obtained in R for differences between test and control at individual time points were uploaded into MetaCore and a workflow data analysis report performed. This generated lists of significantly altered genes that showed changes in expression level at more than one time point. The greatest number of significantly altered genes common between time points was found for 8 hr and 24 hr. The resultant list of common genes between 8 hr and 24 hr was used to build networks of associated pathways and processes. Pathways were sorted for importance by the G-score. The G-score modifies the Z-score (the rank according to saturation of genes from the experiment) based on the number of Canonical Pathways used to build the network. If a network has a high G-score, it is saturated with expressed genes (from Z-score) and it contains many Canonical Pathways. Sorting the table by this value essentially enables you to sort the table by two factors at once.

### Experiment 2: Effect of EPA and AA on gene and protein expression

In order to determine whether the identified changes in gene expression were specific to the n-3 fatty acid EPA, rather than a generic response to the presence of any fatty acid, cells were treated with either EPA, or an n-6 fatty acid (AA), or left untreated (controls). RNA and protein were extracted at a range of time points from 0 to 24 hr and these samples were analyzed by real-time RT-PCR and western blotting, respectively. RNA samples from cells treated with different fatty acids were analyzed for EphB4, Vav2 and EphA1 expression.

### Real-time RT-PCR analysis

RNA samples from EPA, AA and untreated HT29 cells were prepared and analyzed for integrity and concentration as detailed previously. 100 μl reverse transcription reactions were prepared with 2 μg RNA, 2.5 μM polyN (15mer) primer (Operon) and all other reagents were provided from Applied Biosystems TaqMan reverse transcription reagent kit (#n8080234). Reactions were performed in a 96 well plate following manufacturers protocol. cDNA samples were stored at -80°C prior to use.

Primers and probes were designed using the Universal Probe Library Assay Design Centre, by Roche Applied Science. Primers were then purchased from Sigma Genosys and probes from Roche Applied Science. All reactions were prepared using the Corbett robot and run on Applied Biosystems AB One Step Plus under standard conditions with 200 nM each primer, 100 nM probe and Applied Biosystems TaqMan Gene Expression master mix (#4369016) in a total reaction volume of 20 μl. All primer sequences and corresponding probes are shown in Table 1.

**Table 1:** Primer sequences and corresponding probe numbers for real-time RT-PCR analysis

Gene	Forward primer	Reverse primer	Roche Universal Probe Library probe
EphB4	gcccgtcatgattctcaca	gaactgtccgtcgtttagcc	12

EphA1	gcatgaaacgctacatcctg	gtgattcccatctgcgtca	67

Vav2	catcaaggtggaggtgcag	gtacttggcctcggtctcct	67

β-actin	ccaaccgcgagaagatga	ccagaggcgtacagggtag	64

To enable us to compare gene expression changes in this experiment with those identified by microarray analysis in the first experiment, the data for EPA treated cells, normalized using β-actin expression, were then expressed relative to untreated control cell data at the same time point for both data sets, before normalizing to the expression level immediately after media change

### Protein sample preparation and western blotting

Protein extracts were prepared from frozen cell pellets of untreated, EPA treated and AA treated HT29 cells using RiPa lysis buffer (Santa Cruz SC-24948) as per manufacturer's instruction. Samples were stored at -80°C. Pre-stained protein molecular weight marker was purchased from New England Biolabs (P77085). MagicMark western protein standard (LC5600) was purchased from Invitrogen life technologies. Protein concentration of samples was determined using BCA assay kit (#23227) purchased from Thermo Scientific and used as per manufacturer's instructions. NuPage Novex 10% bis-tris gel 1.0 mm 12 well gels, NuPage LDS sample buffer, NuPage transfer buffer, NuPage MOPS SDS running buffer for bis-tris gels and NuPage antioxidant were all purchased from Invitrogen life technologies. 20 μg protein was incubated with 1 μl 0.5 M DTT and 4 × NuPage buffer at 70°C for 10 min. Gels were run following standard conditions at 200 V for 50 min and then transferred to PVDF membrane using the Novex transfer system for 60 min at 30 V.

Mouse monoclonal to Eph receptor B4 (ab70404), rabbit monoclonal to Vav2 (ab52640), rabbit polyclonal to Eph receptor B4 (ab64820) and rabbit polyclonal to beta actin (ab8227) were all purchased from Abcam. Beta actin was selected for use as the loading control to allow direct comparison with the normalized gene expression data from real-time RT-PCR analysis. After transfer, membranes were briefly washed in superblock T20 (TBS) blocking buffer (Thermo Scientific) and blocked for either 60 min at room temp or overnight at 4°C. Primary antibodies were incubated overnight at 4°C at appropriate concentrations. Membranes were washed 4 times in Tris buffered saline and then incubated with appropriate horseradish peroxidase (HRP) conjugated secondary antibody (HRP conjugated anti-mouse antibody (NA931) and HRP conjugated anti-rabbit antibody (NA934) both from GE healthcare). Bands were detected with Amersham ECL plus western blotting detection reagents and visualized using a BioRad Fluor-S Multimager. Blots were stripped for re-probing with Re-Blot plus (Chemicon International) following manufacturer's standard protocol.

### Statistical Analysis

All statistical analysis apart from the array analysis (discussed above) was conducted using the Minitab Package (version X1). Gene expression data is expressed as log2 mean values with standard deviations represented on these plots in proportion to the logged value based on the standard deviation of the unlogged mean. Protein data is expressed as mean and standard deviation. Two-way analysis of variance using the General Linear Model and Tukey post-hoc tests were carried out on logged gene expression data and unlogged protein data to examine the effects of treatment over time. Each time point and each treatment was treated as an independent variable.

## Results

### Experiment 1: Microarray analysis

In total 48 arrays were analyzed in R, 335 genes were identified as significantly altered between EPA treated and untreated control cells at T = 8 hr and more than 500 genes were identified as significantly altered between EPA treated and untreated control cells by 24 hr. The number of significantly altered genes at each time point is shown in Table 2 and the list of genes identified as significantly modified are shown in Additional file 1, Table S1. It is beneficial to maximize the information that can be gleaned from array data sets by looking for genes with similar ontology, or that are involved in the same biochemical pathway [22]. To this end the R analyzed data was uploaded into GeneGo MetaCore™ for network and pathway analyses [23] and identification of shared gene ontologies (GO processes) that were significantly altered by EPA treatment.

**Table 2:** The number of significantly altered genes between EPA treatment and untreated cells as identified by microarray

Timepoint	Number of significantly altered genes	
	
	p < 0.05	p < 0.01
T = 0 hrs	3	1

T = 1 hrs	226	48

T = 2 hrs	1	1

T 4 hrs	102	37

T = 6 hrs	34	15

T = 8 hrs	335	38

T = 24 hrs	543	140

The number of genes identified by microarray and analyzed in R, including Benjamini and Hochberg multiple test correction, as significantly altered between EPA treated and untreated HT29 cells over time.

GeneGo MetaCore identified 155 genes that were significantly altered at both 8 hr and 24 hr and with these a list of 30 networks were identified. The highest scoring network identified by GeneGo MetaCore was for the Ephrin receptor (p < 7.00 × 10^-11^) (Table 3). Additional file 2, Figure S1 shows the simplified Ephrin receptor network created in MetaCore. Pathway analysis revealed significantly altered pathways for T = 8 hr and T = 24 hr. The top ten significantly modified pathways are shown in Additional file 3, Table S2. The Ephrin pathway was fourth ranked most significantly altered pathway identified and from this three key genes EphB4, EphA1 and Vav2 were identified on as having significantly altered gene expression p < 0.05 as a result of EPA treatment (Table 4 and Additional file 4, Figure S2).

**Table 3:** The highest G-score networks and corresponding processes identified using MetaCore [50]

Networks	GO Processes‡	Significantly changed genes involved	p-value	G-score
Ephrin-B receptors, Ephrin-A receptors, Connexin 32, Twinfilin, FXR1/2	cell projection organization and biogenesis (43.8%; 1.652e-19) cell projection morphogenesis (43.8%; 1.723e-19) cell part morphogenesis (43.8%; 1.723e-19) cell morphogenesis (45.8%; 1.974e-18) cellular structure morphogenesis (45.8%; 1.974e-18)	**Receptors: **Ephrin-B receptors ↑; Ephrin-B receptor 4 ↑; Ephrin-A receptors ↑ **Regulators: **Vav2 ↑ **Binding Protein: **Twinfillin ↓; Alpha Actin ↑ **Channels: **Connexin 32↑ **Protein: **FXR1/2 ↑; VAMP4 ↑ **GTPase: **CDC42 ↑	7.00e-11	91.44

FXR, FXR/RXR-α, GIPC, SOX3, Pitpnm (NIR2)	V(D)J recombination (6.8%; 5.712e-06) response to stimulus (56.8%; 1.109e-05) B cell homeostatic proliferation (4.5%; 2.286e-05) organ morphogenesis (25.0%; 2.353e-05) bile acid metabolic process (6.8%; 4.487e-05)	**Transcription Factor: **SOX3 ↑; TITF1 ↑↓; FXR ↑ **Receptors: **Syndecan-1 ↓ **Binding Protein: **Fetulin-A ↑; Pitpnm (NIR2) ↑↓; GIPC ↑; SYNE2 ↑; Actin ↑; RAD23A ↑↓ **Channels: **Kir3.3 ↑ **Transporter: **SLC6A6 ↑↓; Glut1 ↓ **Protein: **COQ4 ↑ **Lipid Kinase: **P3C2B ↑; PI3K class II ↑	3.08e-23	36.22

SET1A, Autophagin-1, ASNS, ETV6(TEL1), POP1 (RNase P/MRP subunit)	tRNA catabolic process (7.7%; 2.622e-06) tRNA 5'-leader removal (7.7%; 7.859e-06) ncRNA catabolic process (7.7%; 1.570e-05) autophagy (11.5%; 2.111e-05) intracellular transport (30.8%; 1.049e-04)	**Transcription Factor: **ETV6 (Tel1) ↑ **Binding Protein: **PEX3 ↑;FXR 2↑; Histone H2A ↑ **Protease: **Autophagin-1↑ **Enzyme: **GSHB ↑; ASNS ↑; Set1A ↑; POP1 (RNaseP/MRP subunit) ↑ **Protein: **ES1 ↑	1.66e-20	35.43

NF-kB, Large 39 S subunit, MRPL16, 2'-5'-oligoadenylate synthetase, TIEG	response to UV (27.3%; 3.661e-10) nucleotide-excision repair, DNA damage removal (18.2%; 3.547e-08) nucleotide-excision repair, DNA incision (13.6%; 1.320e-07) DNA catabolic process, endonucleolytic (13.6%; 1.978e-07) response to light stimulus (27.3%; 2.314e-07)	**Transcription Factor: **TIEG ↑ **Binding Protein: **Fetulin-A ↑; MRPL16 ↓; MRPL37 ↓ XPA ↑; P21 ↑ **Enzyme:**2'-5' oligoadenylate synthetase ↑ SULT1A1 ↑ **Kinase: **PRKX ↑	1.45e-17	35.39

‡ (% refers to objects on network belonging to process; p-value showing statistical significance of the GO process on that network)

**Table 4:** Fold changes of key genes altered after EPA treatment in the Ephrin receptor network.

Gene of interest	Length of treatment (hr)	fold expression	Adjusted P value
EphB4	8	2.2	0.02

	24	1.8	0.02

EphA1	8	2.0	0.02
	24	1.6	0.03

Vav2	8	1.1	0.82

	24	2.2	0.01

Fold changes of key genes identified as significantly altered after EPA treatment of HT29 cells in the Ephrin receptor network. Analysis of microarray data was performed in R as detailed in the materials and methods and includes a Benjamini Hochberg false discovery rate of less than 5%. All data are expressed relative to an untreated control.

### Experiment 2: Effect of EPA and AA on gene and protein expression

**Figure 1:**
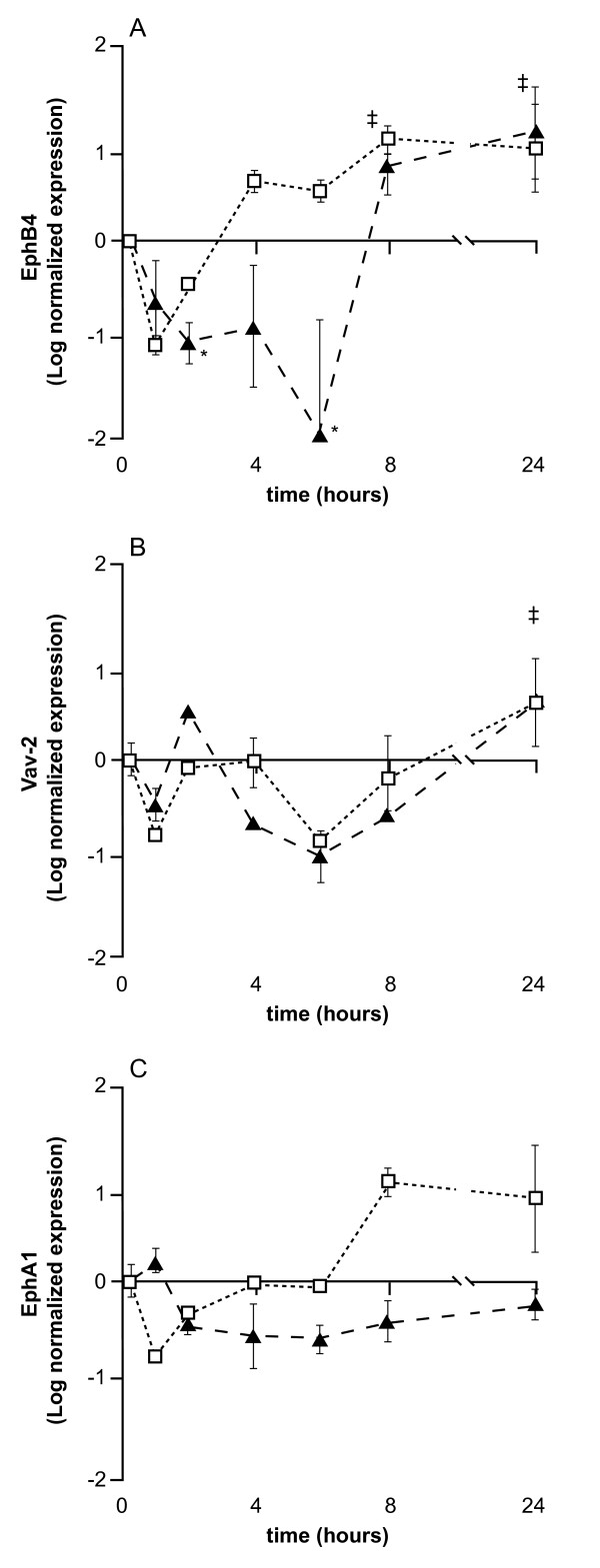
**EPA induced changes in the expression of EphB4, Vav2 and EphA1**. EPA induced changes in the expression of EphB4, Vav2 and EphA1 in HT29 cells as determined by microarray and real-time RT-PCR (TaqMan) over a 24 hr time course in two separate experiments. Data are expressed as log fold change relative to untreated control cells at the same time point. All data is normalized to the initial sample taken immediately after changing the media (< 20 min.). Open circles denote microarray data and filled triangles denote real-time RT-PCR TaqMan data. Each data point depicts the mean of 2-4 flasks of cells. The error bars represent standard deviation with significant difference relative to untreated cells (p < 0.05) represented by * for TaqMan data and ‡ for microarray data. (A) EphB4 is up-regulated in response to EPA treatment at 8 and 24 hr by more than 2 fold. B) Vav2 is up-regulated in response to EPA treatment as determined by real-time RT-PCR analysis at 24 hr by both microarray analysis (p < 0.05) and real-time RT-PCR (p < 0.02 - relative to expression between 1-6 hr). C) EphA1 is up-regulated at 8 and 24 hr in the first experiment using microarray analysis but not in the second experiment using real-time RT-PCR.

Increased expression of EphB4 and EphA1 relative to untreated control cells was seen at both 8 hr and 24 hr and Vav2 expression was also up-regulated after 24 hr exposure to EPA as measured by microarray analysis (Figure 1). The observed changes in expression of Vav2 were very similar in both the first experiment using microarray analysis and in the second experiment using real-time RT-PCR. Similarly, at 8 hr and 24 hr EphB4 expression was similar in both experiments but not at the time points immediately following media change. Although EphA1 showed increased expression in response to EPA in the first experiment, this was not seen in the second.

**Figure 2:**
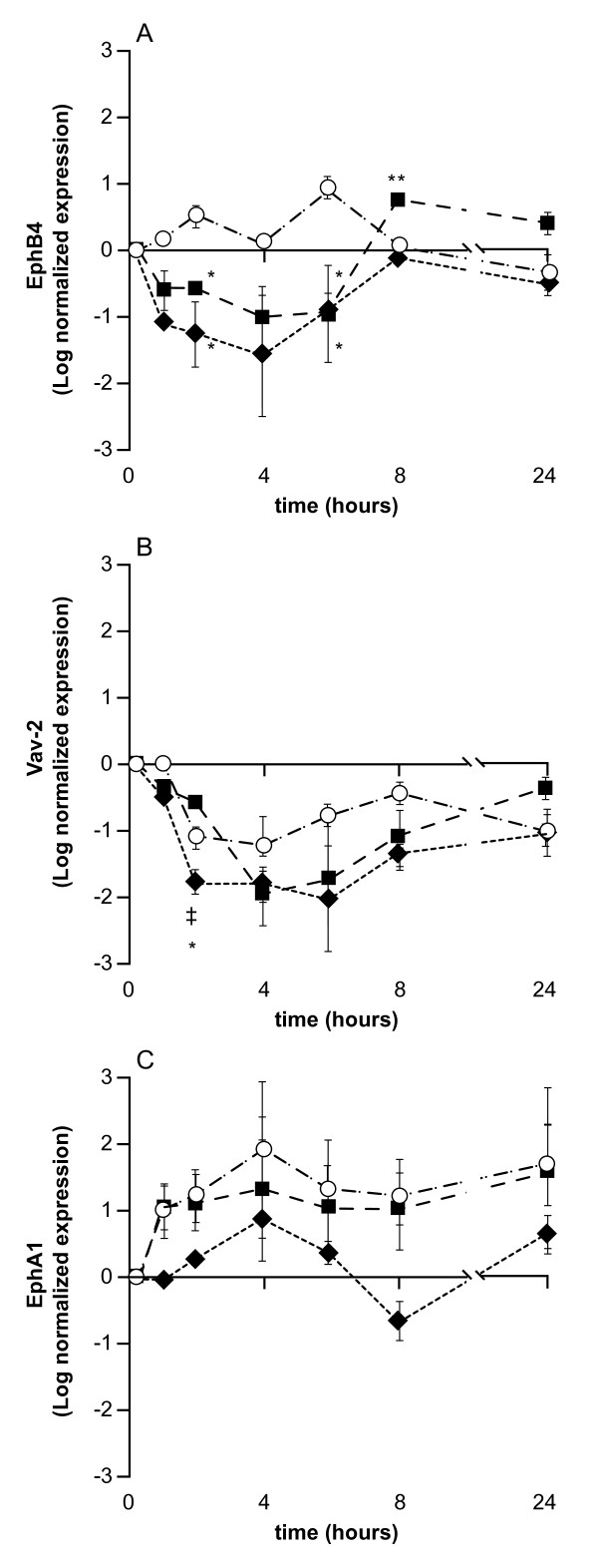
**Effects of EPA and AA on EphB4 gene, Vav2 and EphA1 expression in HT29 cells as determined by real-time RT-PCR analysis**. All data are expressed relative to β-Actin, allowing comparison to figure 3. Each data point represents the log of the mean and standard deviation of three replicate flasks of cells which were untreated, treated with EPA or AA over a 24 hr time course. (EPA closed squares, AA closed diamonds and untreated open circles). Significantly different values are represented by * (comparing EPA to untreated); ** (comparing EPA to AA). ‡ (comparing AA to both untreated and EPA). A) EphB4 expression is significantly affected by time (p < 0.02) and treatment p < 0.001). Post-hoc tests show significant treatment effect for AA (p < 0.001) and not EPA (p = 0.06). Individual time point analysis showed EPA significantly up-regulated EphB4 at 8 hr relative to AA (p = 0.05) and untreated (p = 0.09) and at 24 hr expression was still elevated but not significantly. B) Vav2 expression is significantly affected by time (p < 0.001) and treatment (p = 0.003) and the effect was associated with AA (p = 0.002). AA treatment reduced expression levels at 2 hr as compared to EPA (p = 0.005) and untreated (p = 0.05) but EPA treatment was non-significant compared to untreated cells (p = 0.1). At 24 hr Vav2 expression was higher in EPA treated than untreated cells but not significantly (p = 0.4). C) EphA1 expression is significantly affected by time (p = 0.01) and treatment (p < 0.001). At 4 hr there is a significant increase in gene expression (p = 0.006) and again at 24 hr (p = 0.01). This is associated with decreased expression of EphA1 in response to AA compared to EPA (p = 0.002) and untreated cells (p < 0.001) over the time course, however at individual time points no significant effect was detected.

The effects of different fatty acids on gene expression are shown in figure 2. Analysis of the complete data set for EphB4 using two-way ANOVA showed a significant effect of treatment (p < 0.001) and time (p < 0.02). The effect of time in relation to EphB4 expression was only weakly significant for untreated cells (p = 0.09) but was significant for EPA (p = 0.005) and AA (p = 0.009). Comparison of different treatments at each time point using ANOVA showed that expression of EphB4 was increased following EPA treatment compared to AA treated cells (p = 0.04) at 8 hr post treatment, and weakly significantly compared to untreated control cells (p < 0.09). This effect was still apparent at 24 hr although this did not quite achieve statistical significance compared to controls. Analysis of data over time for each treatment group showed that EPA and AA treatment appeared to produce similar gene expression profiles for EphB4 at time points up to 6 hr. EPA led to an initial reduction in expression at 4-6 hr followed by increased expression at 8 hr (p = 0.02 comparing t = 4 hr and t = 6 hr with t = 8 hr post EPA exposure) AA treatment was associated with a drop in EphB4 expression at t = 4 hr (p = 0.01) relative to t = 8 hr.

Analysis of the complete data set for Vav2 gene expression using two-way ANOVA showed a significant effect of time (p < 0.001) and treatment (p = 0.003). Overall the expression of Vav2 decreased following media change, suggesting a generalized stress response to this process. All treatments showed a significant time dependent effect (untreated p = 0.003, AA treated p < 0.001 and EPA treated p = 0.003). Comparison of treatments at each time point showed that at 2 hr AA treated cells had lower expression levels than EPA treated cells (p = 0.005) and untreated cells (p < 0.05) but otherwise the profiles were all very similar.

No significant differences in gene expression were shown for EphA1 by EPA, AA or untreated cells across the time course. EphA1 was therefore omitted from further investigation by western blotting.

**Figure 3:**
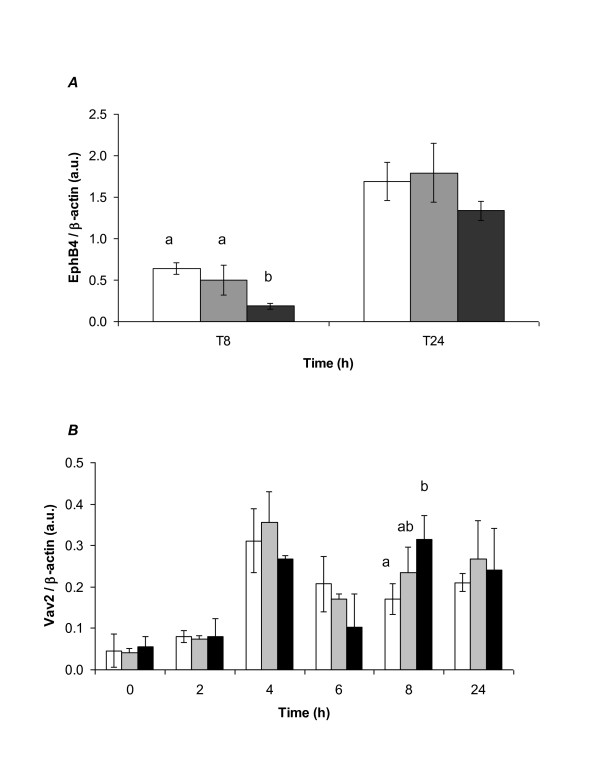
**EPA treatment of HT29 cells leads to significant changes of EphB4 and Vav2 protein expression**. EPA treatment of HT29 cells leads to significant changes of EphB4 and Vav2 protein expression levels as determined by western blotting. Densitometry measurements were taken on a Bio-Rad multi-imager and all data is expressed relative to the house-keeper protein, β-actin, allowing direct comparison of protein expression data to gene expression data as shown in figure 2. EPA treated cells are represented by black bars, AA treated cells by grey bars and untreated cells by white bars. Data are presented as the mean and SD of n = 3 (n = 2 at 4 hr). A) EphB4 protein was not detectable until 8 hr after media change. At 8 hr EPA treatment led to lower protein levels compared to both untreated and AA treated HT29 cells (p = 0.007 and p = 0.034 respectively). There was a highly significant increase in protein levels between 8 hr and 24 hr in all treatment groups (p < 0.005). B) Vav2 protein expression was significantly up-regulated after 4 hr irrespective of treatment (p < 0.05). At 8 hr levels of Vav2 protein were significantly elevated by EPA treatment as compared to untreated control cells (p = 0.043).

Protein samples from cells treated with EPA, AA or untreated cells were analyzed by western blotting for EphB4 and Vav2 expression and normalized to β-actin (Figure 3). Up to 8 hr no EphB4 protein was detectable. This is consistent with a very low abundance of the gene product particularly at the early time points. At 8 hr protein was detectable but expression in cells treated with EPA was significantly lower with respect to both untreated (p = 0.007) and AA treated cells (p = 0.034). By 24 hr there was still a trend for EPA treated cells to show a slight down-regulation in EphB4 expression as compared to both AA and untreated cells but this was no longer significant. In fact, the up-regulation in expression for all three treatments between 8 hr and 24 hr was highly significant (p < 0.005 in all cases). Thus there is an effect of 'time since media change' or 'length of cell culture' on top of which we see the down-regulation of EphB4 protein by EPA.

Vav2 protein expression was significantly up-regulated after 4 hr irrespective of treatment (p < 0.05). At 8 hr levels of Vav2 protein were significantly elevated by EPA treatment as compared to untreated control cells (p < 0.05). EPA treatment led to an apparent increase in Vav2 protein levels as compared to AA treatment but this was not significant. By 24 hr the significant increases in protein levels induced by EPA treatment had diminished and there was no difference between treatments.

There was no apparent correlation between gene expression and protein levels even taking into account the expected delay between changes in gene expression and increased or decreased protein levels.

## Discussion

Although it is well documented that n-3 PUFAs may have a range of health benefits including improved cognitive function [24] and suppression of carcinogenesis [25], the mechanisms by which these effects are mediated are still far from clear. However, PUFAs are known to affect gene transcription by a number of routes that remain imperfectly understood [26]. In order to obtain new insight into potential novel signaling pathways by which PUFAs may act, we investigated changes in gene expression in MetaCore following exposure to EPA using microarray analysis. GeneGo MetaCore analysis of the data was performed to identify pathways modulated by EPA treatment to try and clarify mechanisms by which a high fish diet may exert benefit. The highest scoring network identified by GeneGo MetaCore was for the Ephrin receptor. This network is particularly associated with cell morphogenesis including factors associated with cytoskeletal structure, formation of cell contacts and adhesion to the extracellular matrix; all factors important in both cancer prevention and cognitive function. Three key genes were identified by microarray, EphB4, EphA1 and Vav2, on the associated pathway as having significantly increased gene expression as a result of EPA treatment. These three genes were chosen for further gene expression analysis by real-time RT-PCR and protein analysis by western blotting in order to substantiate the array findings. The Eph receptor family is one of the largest groups of receptor protein kinases [27]. The Ephs (receptors) and their ligands (ephrins) can be divided into two subclasses, A and B, on the basis of sequence homology, structure and binding affinity [28]. Altered expression of Eph receptors and their ephrin ligands has been reported in a large variety of human cancers including epithelial cancers from the colon and ovary [29-33].

Altered expression of Eph receptors and their ephrin ligands has been reported in a large variety of human cancers including epithelial cancers from the colon and ovary [29-33]. This was therefore an area that we were interested to look at to determine whether treatment with EPA could result in perturbation of these pathways and to elucidate whether the mechanism by which high fish consumption is associated with a reduced incidence of colorectal cancer could be via Eph receptor pathways. The apparent increase in EphA1 expression seen in the arrays experiment as a result of EPA treatment was not replicated in the second experiment and so no further analysis of this gene and protein were undertaken. However, EphB4 gene expression was significantly higher in EPA treated cells than in untreated cells by 8 hr after media change but EphB4 protein expression was significantly down-regulated at this time point with respect to both untreated and AA treated HT29 cells. This apparently contradictory result could be explained by the observation that at earlier time point's treatment with EPA led to a down-regulation in EphB4 expression as compared to untreated cells. Allowing time for this to be translated into protein production the down-regulation in EphB4 expression by EPA treatment at 8 hr is expected. However, the AA data do not support such an explanation as this is even more down-regulated initially but there is no difference in protein expression. These data suggest that EphB4 protein is not well correlated with the mRNA levels for this gene and we postulate that there must be a considerable degree of post-translational control.

In the intestinal epithelium EphB receptors are Wnt signaling target genes that control cell compartmentalization along the crypt axis [34]. EphB4 expression in normal healthy colon is low and outlines the membranes of intestinal 'precursors' at the base of the crypt. It has also been found to be consistently over-expressed in tumor cells of early adenomas when compared to normal tissue [30,35], with expression shown to be high (50-100% positive cells) in dysplastic aberrant crypt foci and small adenomas. This high level of expression is shown to be lost during colorectal cancer progression at the adenoma-carcinoma transition and was absent in advanced colorectal tumors [36]. This down-regulation during cancer progression was also observed for EphB2. This would suggest a role for EphBs as a tumor suppressor. The causal role of EphB silencing in colorectal cancer progression is supported by the observation that a reduction in EphB activity exacerbates colorectal tumorigenesis in *Apc*^Min/+ ^mice [36]. It has also been reported that EphB2 has a role in the maintenance of normal tissue architecture in the prostate and mutational inactivation is present in a significant fraction of prostate tumors, suggesting a role for EphB2 in the progression and metastasis of prostate cancer [37]. Thus it is feasible that EPA might suppress carcinogenesis by preventing the down-regulation of EphB activity, although this may well not be directly as a consequence of modified gene expression. The different expression levels of the EphBs in tumor progression and our observed inconsistency between gene and protein expression, highlight the complexity of the pathways involved. Initially increased expression and activation of the pathways appear to benefit tumor growth but ultimately this increased expression imposes restrictions on subsequent tumor progression [36]. Interestingly, it has recently been reported that tumor cells expressing EphB receptors were restricted to large homogeneous clusters by the ligands ephrin-B1 and ephrin-B2 [38]. This response was strongest for ephrin-B1 +EphB2 and ephrin-B1 + EphB3 with the response from ephrin-B1 and EphB4 only producing minor modifications in cell distribution and compartmentalization in the DLD1 colorectal adenocarcinoma and Co115 colon carcinoma cells lines used in that study. This cell compartmentalization subsequently led to a suppression of tumor progression beyond the earliest stage. HT29 cells have very low levels of EphB3 and EphB2 [36], whether the increase in EphB4 protein expression after exposure to EPA was sufficient to induce the compartmentalization response would require further work However this could suggest a mechanism by which the increase in EphB4 protein expression after exposure to EPA in HT29 cells might be beneficial in terms of halting cancer progression from adenoma through to invasive carcinoma.

The routes by which PUFAs might mediate EphB4 expression remain a matter of conjecture. Transcriptional regulation analysis in MetaCore gave no clear evidence of any one particular transcription factor being involved in the control of gene expression by the tested PUFAs in this study (data not included), but it is recognized that EphB4 expression is regulated by one of the HOX family of proteins (HOXA9) and in turn these have been shown to be involved in signaling by other lipophilic molecules, namely steroids and retinoic acid [39].

Vav2 protein expression increased over the 24 hr post media change for all treatments while gene expression initially dropped relative to the initial time point. This suggests that Vav2 expression is sensitive to the stress associated with media change. Protein expression was also higher at 8 hr in EPA treated cells but again these changes were not reflected at the gene transcript level. Vav2 is the second member of the Vav oncogene family and unlike Vav1, which is restricted to hematopoietic cells, Vav2 has been found to be expressed ubiquitously [40]. It is a Rho family guanine nucleotide exchange factor and Vav2 has been shown to have a role in growth factor signaling to the cytoskeleton [41] and is required for integrin-dependant activation of Rac during cell spreading [42]. It has recently come to light that in breast cancer tissue the levels of Vav2 expression as compared to normal or hyperplasic epithelium are down-regulated [43]. Vav2 acts downstream of the Eph A and B receptors in the receptor pathway and its perturbation in breast cancer progression is very relevant to the work we undertook in HT29 cells. Vav2 is important in mediating cytotoxic lymphocyte activity [44] against target cells including cancer cells, but studies in epithelial cells using siRNA knock-down of Vav2 suggest an important role in stimulating cell migration [45]. It may therefore be that one of the mechanisms by which a diet high in n-3 PUFAs may inhibit the development of colorectal cancer is by preventing the down-regulation of Vav2 levels and thereby limiting cancer progression.

In general we saw little difference in response to EPA and AA over the first 6 h after changing the media, although the response to AA was if anything stronger than to EPA for both EphB4 and Vav2. These changes closely follow the changes in redox status mentioned previously and so may be a response to a transitory increase in oxidative stress. However, this association is by no means conclusive and the observed effects could equally well be associated with similar affinities for some nuclear receptors, for example RXR [46]. Alternatively the early transitory responses may reflect the disappearance of EPA from the medium. Peak intracellular concentrations of EPA are found at 6 h [25], and thus it may be that we see an initial short-term exposure effect, followed by a more subtle response to the smaller but more sustained differences in PUFA content found after 8-14 hr. At these later times, responses to EPA and AA were dissimilar, such that the expression of both the EphB4 and Vav2 genes was generally higher in those cells exposed to EPA n-3 PUFA than in those exposed to AA n-6 PUFA.

## Conclusions

Although these studies have been conducted in a tumor cell line, the observation that EPA can change expression of EphB4 may have wider implications and should be investigated further in a wider range of biological systems. For example, the recognized role of Eph receptor signaling in neuronal development [47] may provide at least a partial explanation for the suggested benefits of these PUFAs in relation to cognitive function [48,49]. The health benefits associated with the consumption of very long chain PUFAs of the n-3 series are as yet poorly understood at the molecular level. This study suggests a novel mechanism by which these fatty acids may mediate a wide range of health benefits including limiting cancer progression through modulation of the Eph receptor pathways.

## Competing interests

The authors declare that they have no competing interests.

## Authors' contributions

JFD performed all the experimental work, was involved with the design and discussion of the work and preparation of the manuscript. JJE & RME set up the in house microarray facility, assisted with microarray experiments and submitted microarray data to ArrayExpress. RJF & JS performed the statistical analysis, ITJ was involved in the discussion and design of the experiments. EKL initiated the work, was involved in the discussion and design of the experiments and preparation of the manuscript. All authors have read and approved the final manuscript.

## Supplementary Material

Additional file 1 **Table S1. Table of significantly altered genes as identified in R from microarray**. A table showing all the significantly modified genes as identified in R from microarray data at each time point along with their corresponding p-value, adjusted p-value, GenBank ID, Unigene ID and a gene description. Click here for file

Additional file 2 **Figure S1. Simplified Ephrin receptor network from MetaCore**. The Ephrin receptor network as constructed by MetaCore showing genes significantly altered by EPA treatment of HT29 cells as determined by microarray analysis in R. Click here for file

Additional file 3 **Table S2. List of top ten significantly modified pathways as identified in MetaCore**. A table showing the top ten ranked pathways as identified in MetaCore along with their corresponding p-value Click here for file

Additional file 4 **Figure S2. Cell Adhesion and Ephrins Signalling Pathway from MetaCore**. A pathway map from MetaCore showing the Cell Adhesion and Ephrin signalling pathway which contains many significantly modified genes as a result of EPA treatment as determined by microarray analysis. Click here for file
