# Supplementary material for: Identification of the Eph receptor pathway as a novel target for eicosapentaenoic acid (EPA) modification of gene expression in human colon adenocarcinoma cells (HT-29)
Source: Nutr Metab (Lond). 2010 Jul 12;7:56. doi: 10.1186/1743-7075-7-56 (PMC2912917; doi:10.1186/1743-7075-7-56)
Supplement: Additional file 2 — Figure S1. Simplified Ephrin receptor network from MetaCore. The Ephrin receptor network as constructed by MetaCore showing genes significantly altered by EPA treatment of HT29 cells as determined by microarray analysis in R. [file 1743-7075-7-56-S2.PDF]

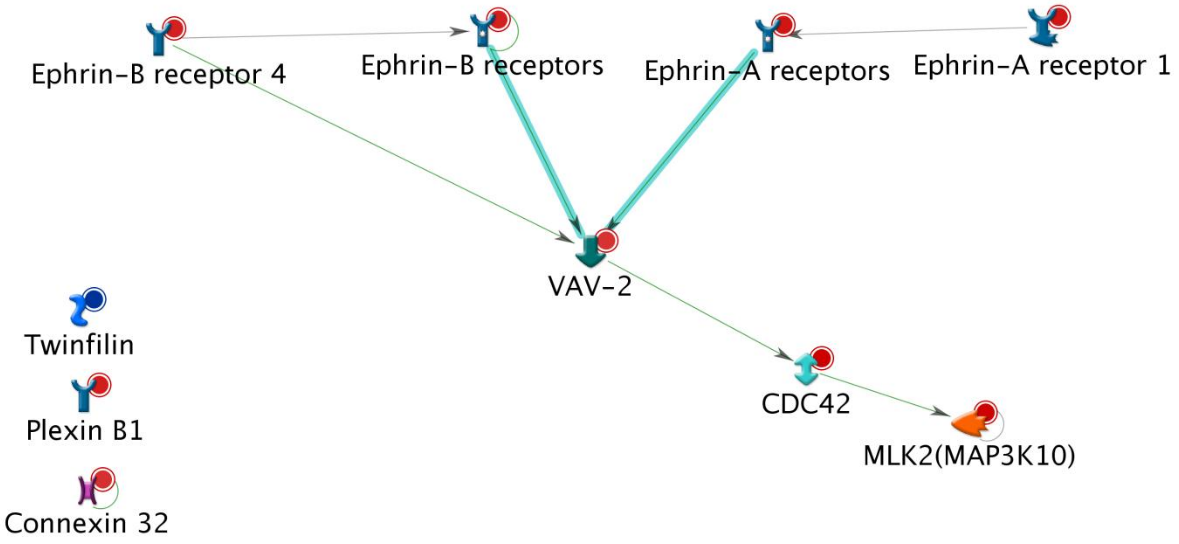

Additional file 2 Figure S1 Simplified Ephrin receptor network from MetaCore

The Ephrin receptor network as constructed by MetaCore showing genes significantly altered by EPA treatment of HT29 cells as determined by microarray analysis in R. Red circles denote genes which are up-regulated by EPA treatment and blue circles represent genes that are down regulated by EPA treatment. The links in the network highlighted by the thick cyan line represent canonical pathways in the network. Green links between genes represent positive effects between the genes; the grey arrows represent technical links between the genes. The on any of the symbols represents proteins or compounds physically connected into a complex or related as a group. Further investigation within MetaCore highlighted that the Ephrin-B receptor and Ephrin-A receptor that were significantly modified by the EPA treatment were EphB4 and EphA1 respectively.

Key to symbols: represents a generic receptor; represents a receptor with enzymatic activity; represents a regulator; represents a G-alpha GTPase; represents a generic kinase; represents a generic binding protein and represents a generic channel.
