# Supplementary material for: Identification of the Eph receptor pathway as a novel target for eicosapentaenoic acid (EPA) modification of gene expression in human colon adenocarcinoma cells (HT-29)
Source: Nutr Metab (Lond). 2010 Jul 12;7:56. doi: 10.1186/1743-7075-7-56 (PMC2912917; doi:10.1186/1743-7075-7-56)
Supplement: Additional file 3 — Table S2. List of top ten significantly modified pathways as identified in MetaCore. A table showing the top ten ranked pathways as identified in MetaCore along with their corresponding p-value [file 1743-7075-7-56-S3.PDF]

Additional File 3 Table S2: List of top ten significantly modified pathways as identified in MetaCore

**Rank**	**Pathway Name**	**pValue**
1	Cytoskeleton remodeling_Regulation of actin cytoskeleton by Rho GTPases	0.0004
2	Apoptosis and survival_Anti-apoptotic TNFs/NF-kB/Bcl-2 pathway	0.0022
3	Apoptosis and survival_Lymphotoxin-beta receptor signaling	0.0022
**4**	**Cell adhesion_Ephrins signaling**	**0.0029**
5	Immune response_Fc epsilon RI pathway	0.0052
6	Cytoskeleton remodeling_Alpha-1A adrenergic receptor-dependent inhibition of PI3K	0.0064
7	Immune response_TLR3 and TLR4 induce TICAM1-specific signaling pathway	0.0071
8	Immune response_CD40 signaling	0.0079
9	Muscle contraction_Delta-type opioid receptor in smooth muscle contraction	0.0119
10	Immune response_CD137 signaling in immune cell	0.0147
