# Supplementary material for: Identification of the Eph receptor pathway as a novel target for eicosapentaenoic acid (EPA) modification of gene expression in human colon adenocarcinoma cells (HT-29)
Source: Nutr Metab (Lond). 2010 Jul 12;7:56. doi: 10.1186/1743-7075-7-56 (PMC2912917; doi:10.1186/1743-7075-7-56)
Supplement: Additional file 4 — Figure S2. Cell Adhesion and Ephrins Signalling Pathway from MetaCore. A pathway map from MetaCore showing the Cell Adhesion and Ephrin signalling pathway which contains many significantly modified genes as a result of EPA treatment as determined by microarray analysis. [file 1743-7075-7-56-S4.PDF]

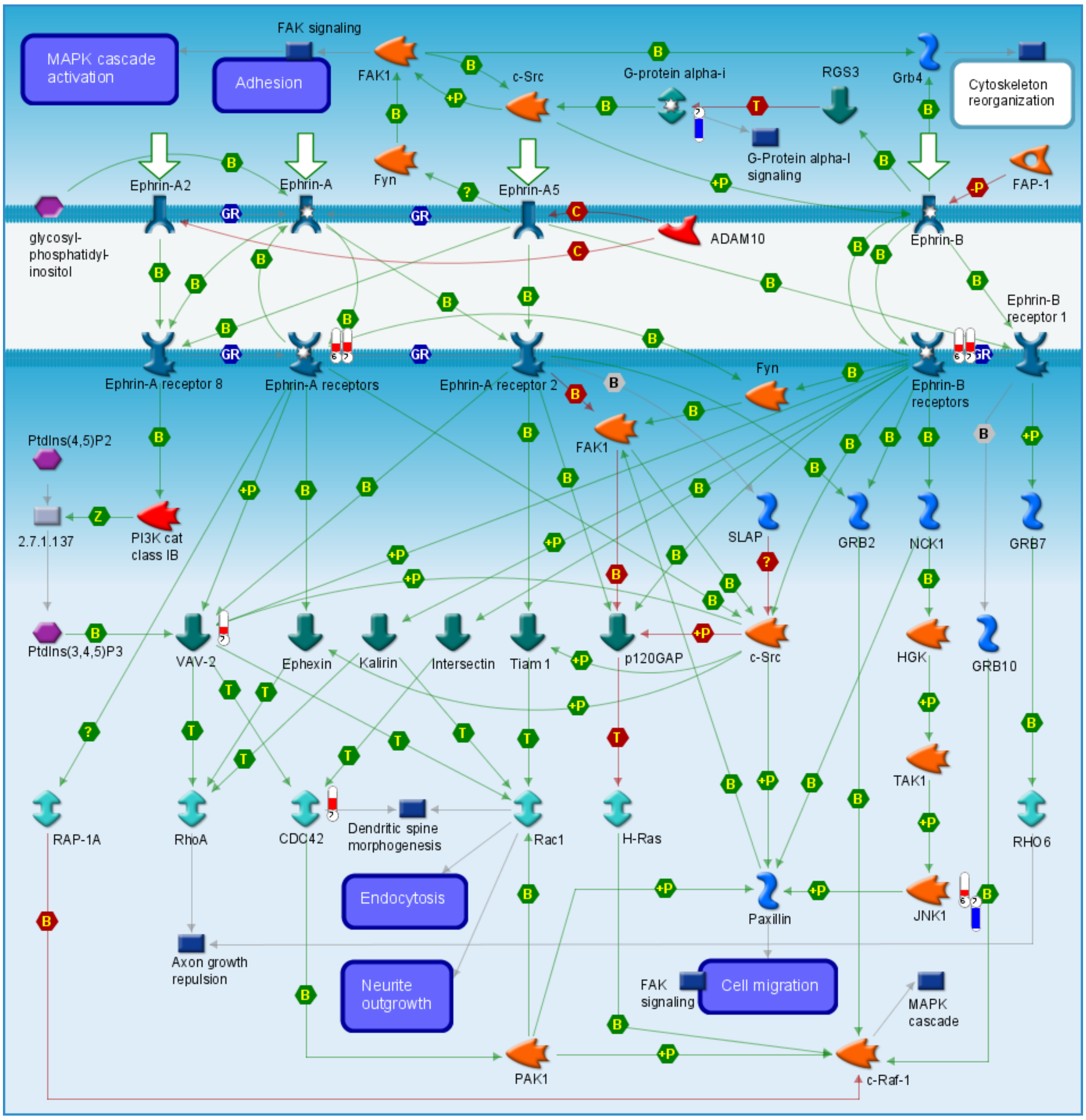


## Additional file 4, Figure S2 Cell Adhesion and Ephrins Signalling Pathway from MetaCore

A pathway map from MetaCore showing the Cell Adhesion and Ephrin signalling pathway which contains many significantly modified genes as a result of EPA treatment as determined by microarray analysis. The thermometers represent significant alterations in gene expression as a result of EPA treatment as determined by microarray. Red represents up regulated genes and blue down regulated genes with the amount of colour demonstrating how much the expression has changed. The numbers associated with the thermometer correspond to the time points, 6 represents T= 8 hr and 7 represents T= 24 hr.

Key to symbols: represents a generic receptor; represents a receptor with enzyme activity; represents a G protein regulator; represents a G-alpha GTPase; represents RAS-superfamily GTPase; represents a generic kinase; represents a lipid kinase; represents a matalloprotease represents a protein phosphatase; represents a generic binding protein

represents a reaction and represents a compound. The on any of the symbols represents proteins or compounds physically connected into a complex or related as a group
